# Effect of flight velocity on droplet deposition and drift of combined pesticides sprayed using an unmanned aerial vehicle sprayer in a peach orchard

**DOI:** 10.3389/fpls.2022.981494

**Published:** 2022-09-29

**Authors:** Longlong Li, Zhihong Hu, Qingju Liu, Tongchuan Yi, Ping Han, Ruirui Zhang, Ligang Pan

**Affiliations:** ^1^Beijing Academy of Agriculture and Forestry Sciences, Beijing, China; ^2^Institute of Quality Standard and Testing Technology of Beijing Academy of Agriculture and Forestry Sciences, Beijing, China; ^3^Research Center of Intelligent Equipment, Beijing Academy of Agricultural and Forestry Sciences, Beijing, China; ^4^National Research Center of Intelligent Equipment for Agriculture, Beijing, China

**Keywords:** unmanned aerial vehicle (UAV), spray deposition, spray drift, combined pesticides, canopy, peach orchard

## Abstract

Extensive research has been conducted on plant protection unmanned aerial vehicle (UAV) chemical application technology in recent years owing to its importance as a means of pest and disease control. UAV spraying in orchards faces the drawback of drift risk and can be hazardous to non-targeted crops, humans, and the environment. A detailed and systematic analysis must be performed to determine the uniformity and drift risk of plant UAV sprays. In this study, a peach orchard is sprayed with a plant-protection UAV at three different flight velocities and we evaluate the combined pesticide deposition performance of the canopy, ground loss, downwind ground drift, and airborne drift. Additionally, the droplet size and coverage rate in the canopy are calculated by using water-sensitive paper. The results demonstrate that there is significant difference in the droplet size at flight velocities of 1–3 m/s. The droplet size in the lower canopy is slightly smaller than those in the middle and upper parts. Increasing the flight velocity helps the pesticide droplets to spread and penetrate the canopy. However, it also causes a non-uniform pesticide deposition, reduced effective coverage ratio and effective density ratio. Among the three pesticides used in the experiment, imidacloprid exhibits the best deposition efficiency. The deposition amount and normalized deposition amount in the canopy were the highest at a flight velocity of 2 m/s, accompanied by a lower ground loss under the canopy. The highest near-field ground drift is observed at a velocity of 1 m/s, and the far-field airborne drift is highest at 3 m/s. Lastly, this study provides a reference for the commercial application of plant-protection UAVs.

## Introduction

Diseases and pests are the primary factors affecting crop production, which includes the yield and quality of grain (Godfray et al., 2010). Statistical data confirm that diseases, pests, and weeds account for 30% of the global crop losses each year (Guo et al., 2019). Therefore, active measures must be taken to reduce the impact of diseases and pests. Currently, the most widely used method for the prevention and control of diseases and pests involves spraying chemical pesticides on crops (Chen et al., 2021; Sparks and Bryant, 2021; Zhang Y.L. et al., 2021). Various methods have been developed to improve the spraying efficiency and control the effect of pesticides, such as ground spraying, aerial spraying, air-assisted spraying, and knapsack spraying (Qin et al., 2016; Pan et al., 2017; Wang et al., 2019b).

Extensive research has been conducted on plant-protection unmanned aerial vehicles (UAVs) in East Asia, China, and other regions in recent years (Li et al., 2019; Chen et al., 2021). In China, plant protection UAVs have been widely implemented for field crops, fruit trees, tea trees, cotton, and several other crops. This method significantly improves the operational efficiency, and reduces the labor and exposure to pesticides when compared to traditional knapsack spraying methods. Additionally, plant protection UAVs can overcome the limitations of traditional ground spraying equipment and can also realize free pesticide application operations in hills, mountains, and paddy fields. Furthermore, the downwash airflow generated by the rotors can overturn the leaves and promote the penetration and attachment of fine droplets inside the canopy (Meng et al., 2019). These advantages have led to the increased application of plant-protection UAVs.

Droplet deposition and spray drift characteristics are important indicators for the evaluation of plant protection equipment. Extensive research has been conducted on the factors affecting droplet deposition in UAV spraying, such as droplet size (Chen et al., 2020), flight velocity (Meng et al., 2020; Zhang S.C. et al., 2021), flight height (Zhang S.C. et al., 2021), tree shape (Pan et al., 2017; Tang et al., 2018; Meng et al., 2020), wind field (Chen et al., 2017), spray volume (Wang et al., 2019a; Li et al., 2021b), aerial spray adjuvants (Meng et al., 2018), UAV type (Wang et al., 2017, 2021), and nozzle type (Wang et al., 2021). In terms of crop types, the existing studies are primarily focused on field crops. The effects of UAV parameters on droplet deposition in wheat (Lou et al., 2018; Shan et al., 2021), cotton (Qin et al., 2018), and rice (Chen et al., 2020), corns (Zheng et al., 2017) were analyzed extensively. The canopy of fruit trees is three-dimensional and the density of branches and leaves is higher when compared to field crops. Overcoming these limitations and improving the droplet deposition uniformity in the canopy is an important research objective for plant-protection UAVs. Chen et al. (2017) and Tang et al. (2018) analyzed the effects of flight velocity, height, and application rate on droplet deposition and distribution in citrus canopies.

The existing studies on spray tests in orchards with UAV sprayers primarily use tracers to simulate pesticides. The droplet deposition on the target and the spread of the tracer solution may not concur with the results of actual pesticide spraying due to the aerosol characteristics. Additionally, a compound pesticide spraying mode is generally adopted during actual application to avoid various diseases and pests, which increases the uncertainty of deposition.

Peach (Prunus persica) is one of the most popular fruits worldwide and presents several health benefits. Peach trees are particularly vulnerable to pests and diseases (e.g., *Myzus persicae*, *Cercospora circumscissa* Sacc.) at different growth stages (Li et al., 2018; Samad et al., 2019). The spraying of pesticides and fungicides can ensure the quality and yield of peaches. In addition to the advantages of UAV spraying techniques mentioned above, the UAV spraying of chemical pesticides can overcome the drawback of a lack of row spacing for Y-shaped peach trees. However, the research on the canopy deposition of pesticides sprayed by plant-protection UAVs remains limited. Therefore, a detailed and systematic analysis of the spray deposition and drift from a UAV sprayer in a peach orchard is crucial.

In this study, spraying tests were conducted in a peach orchard to obtain a better understanding of the canopy deposition and droplet drift characteristics of plant-protection UAV spraying methods. The spray solutions were prepared by using three commonly applied pesticides. The effects of flight velocity on the canopy deposition and drift were analyzed. The deposition distribution characteristics of insecticides and fungicides in the canopy were also analyzed using ultra-high-performance liquid chromatography-tandem triple quadrupole mass spectrometry. This study can provide data support for the selection and optimization of the pesticide application parameters for fruit trees using plant-protection UAVs.

## Materials and methods

### Experimental plots

The experiments were carried out commercial peach orchard at growth stage BBCH 91 “Shoot growth completed; foliage still fully green” (Meier et al., 1994) located at Dahuashan Town, Pinggu District, Beijing, China. The main peach variety of the orchard is Okubo. The trees were planted at a density of 1,000 trees/ha with a canopy height of 3.5 m, row spacing of 5 m, and between-tree spacing of 2 m.

### Plant protection unmanned aerial vehicle

**FIGURE 1:**
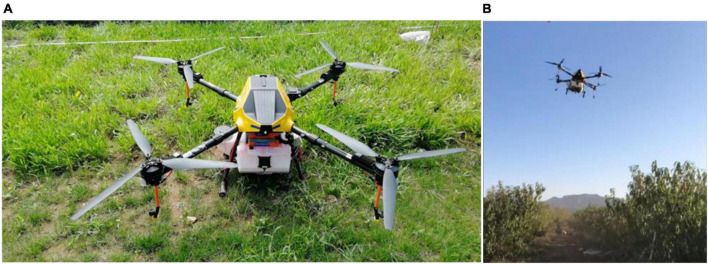
Experimental site. **(A)** 3WYD-4-22A plant protection UAV; **(B)** spraying in peach orchard.

A four-rotor electric plant protection UAV with a spray tank volume of 22 L was used in the experiment (3WYD-4-22A, Wuxi Hanhe Aviation Technology Co., Ltd.), as shown in Figure 1. This UAV is based on a three-blade propeller design which effectively reduces the vibration of the airframe during the spray operation and also improves the flight balance. Additionally, strong downwash airflow can be generated to promote the penetration of droplets into the canopy. A flat-fan nozzle is fixed under each rotor wing. The pesticide application operation mode includes both automatic and manual modes. In the automatic mode, the flight velocity, height, application rate, and route can be set beforehand, and the UAV can implement an autonomous spray operation. Thus, the flight errors caused by manual operation can be effectively avoided. Before conducting the experiment, the effective spray swath of the UAV was determined to be 4.0 m. Table 1 lists the technical parameters of the plant-protection UAV used in this experiment.

**TABLE 1:** Technical parameters of the plant protection UAV used in this study.

Classification	Parameters
Number of rotors	4
Number of nozzles	4
Nozzle type	Flat-fan, Lu120-015
Flow rate (in L/min)	0–7, adjustable
Tank capacity (in L)	22
Size (in m)	1.235 × 1.235 × 0.647
Rotor diameter (in mm)	838
Flight velocity (in m/s)	1.0–7.0
Effective spray swath (in m)	4
Flight duration (in min)	30
Operation efficiency (in ha/hour)	10–14
Positioning mode	GNSS + RTK
Operation method	Intelligent stability control
Weight (in kg)	23.5

### Experimental design

**FIGURE 2:**
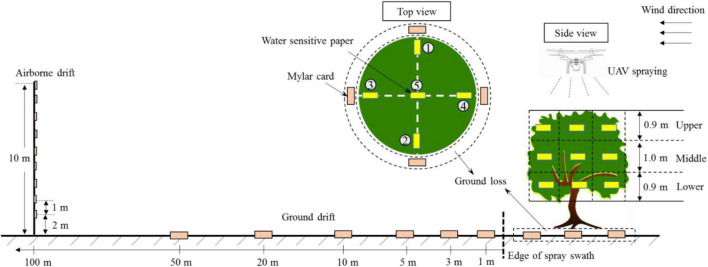
Schematic diagram of sample layout in the treated area.

The experiments were conducted in a field with an area of 150 m × 50 m; the plant protection UAV performed the spraying operations along tree rows with a spraying length of 50 m. The UAV performed one-and-a-half rounds of spraying in each test, covering three adjacent rows of fruit trees. The spray-treated area was 50 m × 15 m. The measurements comprised four parameters: droplet deposition in the canopy, ground loss, ground drift, and airborne drift. Water-sensitive paper (WSP, 26 mm × 76 mm, Syngenta Crop Protection AG, Basel, Switzerland) and a Mylar card (MCD, 85 mm × 54 mm, Wuxi Baike Electronic Materials Co., LTD., China) were selected as the droplet collectors. Figure 2 depicts the sample layout of the experimental area.

#### Canopy deposition

Three typical peach trees were selected as targets in the spray-tested area. The canopy of each peach tree was divided into three layers, i.e., upper, middle, and lower. Each canopy layer was divided into five azimuths based on the UVA flight direction: front, back, left, right, and center positions, corresponding to locations 1, 2, 3, 4, and 5 in Figure 2, respectively. There were 15 sampling points in the target tree. A WPS card was fixed on the leaf using a paper clip at each sampling point to ensure that the sensitive side of the WPS faced upward, which was used to measure the droplet size, coverage, and density in the canopy. Five complete leaves were collected from each sampling point to determine the pesticide content on the leaf surface (mg/kg).

#### Ground loss

During the actual chemical applications, the pesticide droplets are not completely deposited in the canopy. Some of the droplets pass through the canopy and are deposited on the ground, causing soil pollution. Five MCDs were arranged on the ground under the three target peach trees to collect the pesticides lost on the ground. MCDs were placed in five directions under the canopy, similar to the arrangement of the WPS in each canopy layer.

#### Downwind ground drift

The plant-protection UAV adopts the aerial operation mode. The pesticide droplets easily form a downwind drift due to crosswinds, causing pesticide damage to adjacent sensitive crops, along with water pollution, fish and shrimp deaths, and other events. Five MCDs were arranged on the ground in the downwind direction at distances of 1, 3, 5, 10, 20, and 50 m from the edge of the spray swath to measure the drift mass at different distances.

#### Airborne drift

To further understand the drift potential of UAV spraying, a metal pole was fixed at a distance of 100 m from the edge of the treated area. Nine MCDs were successively fixed from bottom to top at intervals of 1 m at a height of 2–10 m from the ground. The card interface was perpendicular to the wind direction.

The flight velocity of the UAV sprayer was set to 1, 2, and 3 m/s, and the relative height between the UAV sprayer and the top of the canopy was set to 2 m. In this study, the application rate was set as 33 L/ha. Therefore, the nozzle flow rate at 1, 2, and 3 m/s were 0.79, 1.58, and 2.37 L/min, respectively. During the experiment, the mean temperature, mean humidity, and mean wind speed of the environment were 16.8°C, 46.3%, and 1.8 m/s, respectively.

After the spraying is completed, the droplets were allowed to dry on the target surface for 5 min, and all samples were carefully collected and stored in ziplock bags. A desiccant must be placed in the ziplock bag to collect the WPS to prevent them from being contaminated by moisture. The collected peach leaves and MCDs were stored in a small refrigerator for further analysis.

### Reagents of combined pesticides

Combined pesticide were prepared by using three widely applied pesticides and fungicides: difenoconazole, azoxystrobin, and imidacloprid. Formulations containing 325 g L^–1^ suspension concentrate of difenoconazole-azoxystrobin (200 g L^–1^ for azoxystrobin and 125 g L^–1^ for difenoconazole) were obtained from Syngenta Nantong Crop Protection Co., Ltd. (Jiangsu, China). This formulation is widely employed to control and prevent peach anthracnose, peach brown spot shot holes, and other diseases. Formulations containing imidacloprid (25%, wettable powder) were obtained from Hebei Kaisite Agrochemical Co., Ltd. (Hebei, China). Imidacloprid wettable powder (WP) is used to control peach aphids, scale insects, and other common pests.

The three analytical standards used in the pesticide deposition detection stage were purchased from Beijing Mindleader Agroscience Co., Ltd. (Beijing, China), with purities of 98.0% for azoxystrobin, 95.0% for difenoconazole, and 97.0% for imidacloprid. Analytical grade NaCl and MgSO_4_ were obtained from Sinopharm Chemical Reagent Co., Ltd. (Shanghai, China). Methanol, acetonitrile, and ammonium acetate were purchased from Thermo Fisher Scientific (Waltham, MA, United States). The primary secondary amine (PSA) and graphitized carbon black (GCB) were provided by Shanghai Aladdin Biochemical Technology Co., Ltd. All the solvents used for measuring pesticide deposition were of the LC-MS grade.

### Sample processing

The water-sensitive paper samples were scanned into digital grayscale images by using a TSN450 handheld scanner (Tiancai Electronics (Shenzhen) Co., Ltd.) with a resolution of 1200 × 1200, and the blue droplet spots on the WPS surface were analyzed by using the *i*DAS image processing software which was developed by the National Research Center of Intelligent Equipment for Agriculture (Xu et al., 2016). This software can be used to quickly calculate the spray deposition parameters, such as the droplet spectral distribution, coverage, and number of droplets. In this study, the droplet coverage rate (Cov), volume median diameter (VMD), diffusion ratio (RD), and droplet density (Dent) of droplets on a WPS were measured.

The pesticide deposition on the peach leaves was recorded in terms of the mass of active ingredient (a.i.) per leaf (mg a.i. per kg biomass). Pesticide recovery on mylar cards in the ground loss, ground drift, and airborne drift tests was of the mass of the active ingredient (a.i.) per unit area of the maylar card (μg a.i. per cm^2^). Based on the application rate, the theoretical deposition of the three pesticides is determined to be 2.00 μg a.i. /cm^2^ for azoxystrobin, 1.25 μg a.i. /cm^2^ for difenoconazole, and 0.75 μg a.i. /cm^2^ for imidacloprid. Ultra-high-performance liquid chromatography-tandem mass spectrometry (UPLC-MS/MS) was used to determine the deposition of the three pesticides from the leaves and droplet collection cards. The deposition amounts of the difenoconazole, azoxystrobin, and imidacloprid agents were represented by DEP_Dif_, DEP_Azo_, and DEP_Imi_, respectively. The UPLC-MS/MS parameters of azoxystrobin, difenoconazole, and imidacloprid were determined before the measurement, and the calibration curves, R^2^, LOD, LOQ, recoveries, and RSD of azoxystrobin, difenoconazole, and imidacloprid in the leaves were developed based on the analytical standards.

The uniformity of the droplet distribution is an important index of the pesticide application quality, which is described by the coefficient of variation (*CV*). The smaller the *CV* value, the more uniform the droplet distribution. The calculation formula is given as:


$$\text{CV}=\frac{S}{\bar{X}}\times 100\%\,S=\sqrt{\frac{\sum_{i=1}^{n}{\left(X\,i-\bar{X}\right)}^{2}}{n=1}}$$

where *CV* denotes the coefficient of variation (%), S denotes the standard deviation of one group, *X* denotes the average deposition data for one group, *n* denotes the number of samplers, and *X*_i_ denotes the deposition from each sampling point.

The droplet size distribution is a parameter which directly reflects the distribution of droplets in the target. When applying pesticides, a relatively uniform droplet size deposit on the leaves achieves better coverage and control. Diffusion ratio, RD, was used to measure the spectral distribution quality of the droplet (Musiu et al., 2019). It represents the uniformity of the droplet size distribution on a WPS. In general, this value exhibits a positive correlation with the uniformity of the droplet size distribution; a larger RD indicates a more uniform droplet spectrum.


$$R\,D=\frac{N\,M\,D}{V\,M\,D}$$

Here, NMD, i.e., the number median diameter, denotes the droplet diameter below which the droplet diameter is 50% of the total number of drops (in μm). VMD, i.e., the volume median diameter, denotes the droplet diameter below which smaller droplets constitute 50% of the total volume (μm).

Droplet deposition penetration in the tree canopy represents the diffusion ability. The penetration efficiency in the vertical direction of the canopy was calculated as follows:


$$P\,E_{V}=\frac{D\,E\,P_{L\,o\,w}}{D\,E\,P_{U\,p\,p\&M\,i\,d}}\times 100\%$$

where PE*_*V*_* denotes the vertical deposition penetration (in %), *DEP*_*Low*_ denotes the mean value of the amount of deposition in the lower canopy (mg/kg), and *DEP*_*Upp&Mid*_ denotes the mean value of the deposition amount collected in the upper and middle canopies (mg/kg).

The diffusion efficiency in the horizontal direction of the canopy was calculated as follows:


$$P\,E_{H}=\frac{D\,E\,P_{i\,n\,t}}{D\,E\,P_{e\,x\,t}}\times 100\%$$

where PE*_*H*_* denotes the deposition penetration in the horizontal direction (in %), *DEP*_*int*_ denotes the mean value of the amount of deposition in the interior zone (mg/kg), and *DEP*_*ext*_ denotes the mean value of the deposition in the exterior zone (mg/kg). Samples 1, 2, 3, and 4 at the periphery of the canopy were set as the exterior zones, and sample 5 was set as the interior zone. The deposition in the canopy improves when this value is closer to 1 (Chen et al., 2022).

The minimum droplet density and coverage rate required for canopy deposition in the traditional ground orchard air-assisted spray mode with an application rate of 500–7000 L/ha, are 30/cm^2^ (Grella et al., 2022) and 10–15% (Deveau et al., 2021), respectively. However, ultra-low volume (ULV) or very low volume (VLV) applications are adopted for plant protection UAV sprays implemented in orchards with an application rate of 45–150 L/ha. The minimum droplet density threshold required for effective spray swath measurement of the plant protection UAV is 15 deposits/cm^2^ (MH/T 1002.1, 2016). Therefore, we selected the effective coverage ratio (ECR) and effective density ratio (EDR) as the two indicators, according to Wang et al. (2022), to evaluate the deposition performance of the plant-protection UAV. ECR represents the ratio of the sample number with a droplet coverage of more than 1% of the total samples, and EDR represents the ratio of the sample number with a droplet density of more than 15 deposits/cm^2^ to the total samples.

The deposition efficiency of the three pesticides could not be compared owing to the difference in the contents of the active ingredients of the three pesticides in the spray tank. Therefore, the measured amount of sediment was normalized to obtain the normalized deposition amounts, DEP_Azo–nor_, DEPD_if–nor_, and DEP_Imi–nor_ (mg⋅cm^2^/kg⋅μg) based on the previously described content of the active ingredients of the three pesticides (2.00 μg a.i. /cm^2^ for azoxystrobin, 1.25 μg a.i. /cm^2^ for difenoconazole, and 0.75 μg a.i. /cm^2^ for imidacloprid).

An ANOVA test was conducted to evaluate the droplet parameters and deposition data at different canopy locations and three flight velocity settings, at a significance level of 0.05. All the statistical analyses were performed using the IBM SPSS Statistics (Version 17.0) software for Windows.

## Results

### The effect of flight velocity on droplet size in the canopy

**FIGURE 3:**
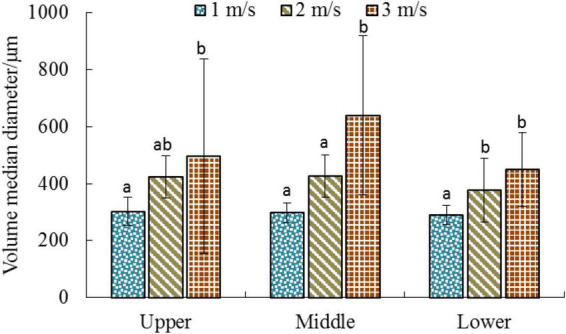
Comparison of droplet volume median diameter on WPS at three canopy layers. Letters indicate significant differences between flight velocities (Duncan test, α = 0.05).

**FIGURE 4:**
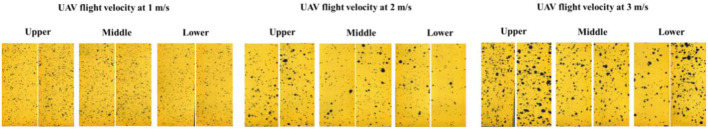
Droplet distribution stains on water sensitive paper in canopy layers under different flight velocity of UAV.

Figure 3 depicts the distribution of the droplet VMD for different combinations of velocity and canopy height. The size of the droplets gradually increases with an increase in the flight velocity from 1 to 3 m/s; this change can be observed by the naked eye, as shown in Figure 4. The blue color represents the distribution of the droplets. The flight velocity significantly affected the droplet size in the same canopy layer. For example, in the upper canopy, the corresponding droplet sizes at flight velocities of 1, 2, and 3 m/s were 302, 423, and 496 μm, respectively, and the droplet size at 3 m/s increased by 64.23% when compared to that at 1 m/s. This was mainly attributed to the downwash airflow generated by the UAV rotor. An increase in the flight velocity leads to the formation of wingtip vortices and other airflow structures. These flow structures drive the movement of fine droplets with the airflow, resulting in an increase in the number of larger droplets deposited on the canopy. The canopy height level does not significantly affect the droplet size. However, the lower canopy droplet size (VMD_Low_) is slightly lower than the upper canopy droplet size (VMD_Upp_) and the middle canopy droplet size (VMD_Mid_), as expected. During the droplet deposition on the canopy, the branches and leaves on the upper layer block the large droplets, while the fine droplets easily pass through the pores of the branches and leaves and settle into the canopy. Particularly, smaller droplets may be required to ensure an effective deposition of pesticides in a canopy with high crown density. For UAVs, the downwash airflow may promote the penetration of droplets into the interior of the canopy. On the one hand, the downwash airflow causes disturbance to the canopy, breaking the original branch and leaf distribution structure, and the porosity of the canopy becomes larger; On the other hand, the downwash airflow increases the movement velocity of the droplets and enhances the kinetic energy of the droplets transported to the canopy.

**FIGURE 5:**
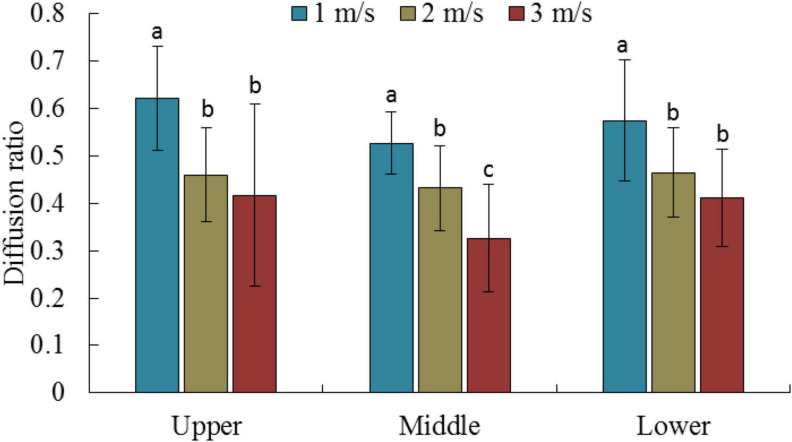
Mean diffusion ratio for different canopy locations and flight velocity. Letters indicate significant differences.

The diffusion ratio is a widely used international index to measure the spraying effect of droplets. It can effectively characterize the uniformity of the droplet diameter distribution on the surface of the target. The ideal value of the droplet diffusion ratio is 1; that is, the volume of all the droplets is identical. Generally, the droplet distribution is considered even when the diffusion ratio range lies within 0.67–1 (Chen et al., 2022). Figure 5 presents the diffusion ratio of the droplets on the surface of the water-sensitive paper at different flight velocities. Unfortunately, the droplet distribution was not ideal for the set experimental conditions and the level of RD > 0.67 was not achieved. The diffusion ratios exhibited significant variation between different velocities at the same canopy height. Overall, the diffusion ratio of the surface of the water-sensitive paper exhibited a gradual decreasing trend with the increase in flight velocity, indicating that an increase in the flight velocity reduces the uniformity of the droplet size distribution. The average diffusion ratios at velocities of 1, 2, and 3 m/s were 0.58, 0.45, and 0.39, respectively, as shown in Table 2. The DR values at different canopy heights did not vary significantly at the same velocity. However, Xu et al. (2017) confirmed that when the plant protection drones perform rice application operations, the diffusion ratio in the middle layer of the rice is better than that in the upper and lower layers.

**TABLE 2:** Spray coverage characteristics in canopy for the different flight velocities.

Parameters	Treatment			
		1 m/s	2 m/s	3 m/s
Upper canopy coverage	Cov_Upp_ (%)	2.25 ± 1.71a	2.98 ±3.10a	3.4 ± 4.34a
Middle canopy coverage	Cov_Mid_ (%)	2.44 ± 1.54a	3.67 ± 3.71a	6.49 ± 10.30a
Lower canopy coverage	Cov_Low_ (%)	1.69 ± 1.51a	2.82 ± 2.30a	4.17 ± 4.38a
Exterior zone coverage	Cov_Ext_ (%)	2.3 ± 1.68a	3.15 ± 3.11a	3.52 ± 6.00a
Interior zone coverage	Cov_Int_ (%)	1.45 ± 0.95a	3.09 ± 4.27a	9.30 ± 8.62b
Mean coverage	Cov_Mean_ (%)	2.13 ± 1.59a	3.14 ± 3.32ab	4.68 ± 6.89b
Coverage distribution uniformity	CV (%)	74.70	105.88	147.49
Effective coverage ratio	ECR (%)	73.30	71.10	57.80
Upper canopy droplet density	Den_Upp_ (deposits/cm^2^)	16.27 ± 13.49a	11.41 ± 9.31a	9.08 ± 10.67a
Middle canopy droplet density	Den_Mid_ (deposits/cm^2^)	18.84 ± 9.17a	13.93 ± 10.81a	12.66 ± 15.80a
Lower canopy droplet density	Den_Low_ (deposits/cm^2^)	13.92 ± 11.81a	11.84 ± 9.82a	13.85 ± 10.38a
Exterior zone droplet density	Den_Ext_ (deposits/cm^2^)	17.43 ± 12.26a	12.54 ± 9.56ab	9.57 ± 11.01b
Interior zone droplet density	Den_Int_ (deposits/cm^2^)	12.01 ± 6.92a	11.83 ± 11.46a	21.06 ± 14.12a
Mean droplet density	Den_Mean_ (deposits/cm^2^)	16.34 ± 11.54a	12.39 ± 9.83a	11.86 ± 12.42a
Effective density ratio	EDR (%)	48.90	33.33	28.90
Upper canopy droplet size	VMD_Upp_ (μm)	302.15 ± 50.17a	423.54 ± 74.33ab	496.92 ± 341.03b
Middle canopy droplet size	VMD_Mid_ (μm)	297.00 ± 35.11a	427.00 ± 74.17a	639.33 ± 278.91b
Lower canopy droplet size	VMD_Low_ (μm)	290.13 ± 33.19a	376.87 ± 113.07b	449.93 ± 129.10b
Mean droplet size	VMD_Mean_ (μm)	301.89 ± 39.58a	420.54 ± 82.35b	512.51 ± 261.30c
Average diffusion ratio	RD	0.58 ± 0.11a	0.45 ± 0.092b	0.39 ± 0.14c

### The effect of flight velocity on droplet coverage characteristics in canopy

**FIGURE 6:**
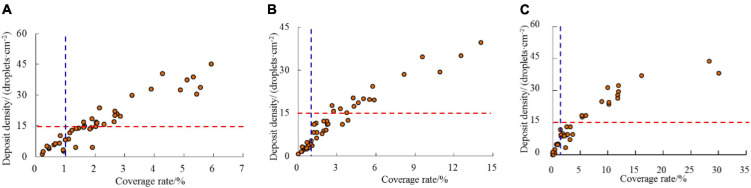
Plots of deposit density and spray coverage rate for different flight velocities. The horizontal red dashed line and the vertical blue dashed line represent the minimum droplet density (15 deposits/cm^2^, Song et al., 2017) and coverage (1%, Chen et al., 2022) required for plant protection UAV application, respectively. **(A)** 1 m/s. **(B)** 2 m/s. **(C)** 3 m/s.

Figure 6 presents the correlation between Cov and Dent under real operating conditions for plant-protection UAVs. Cov and Dent exhibit a good linear correlation, except for a few values with Cov of more than 20% for 3 m/s, which concurs with the findings of Grella et al. (2020). The study reported that a good linear correlation is generally observed between Cov and Dent when the Cov is less than 20% in the measurement of the droplet deposition using water-sensitive paper. This is primarily attributed to the fact that more droplets exhibit overlapping staining when the Cov exceeds 20%. The effect of pest control exhibits a strong correlation with the coverage characteristics of droplets; however, excessive droplet coverage does not indicate high control efficiency (Garcerá et al., 2011). Chen et al. (2013) considered a Cov of more than 30% on water-sensitive paper to indicate excessive spraying. For the conventional orchard air-assisted spraying approach, the effective thresholds of dents for fungicide and insecticide spraying were 70 deposits/cm^2^ and 30 deposits/cm^2^, respectively (Zhu et al., 2011; Salcedo et al., 2020). However, the plant protection UAV adopts an ultralow-volume spray method (ISO 5681, 2020) with less water consumption and a larger concentration of pesticides. Further research is required to determine the consistency of the droplet density and coverage required for disease and pest control with the conventional spraying methods. However, in China, the minimum value of Dent required for the effective spray amplitude of the current plant protection UAV is observed to be 15 deposits/cm^2^ based on a large number of spraying experiments conducted in the early stage; relevant standards have also been formulated for regulation (MH/T 1002.1, 2016; Song et al., 2017). Therefore, droplet densities higher than 15 deposits/cm^2^ and coverage rates higher than 1% (Wang et al., 2022) were selected as the effective deposition thresholds. For the three flight velocities, the number of samples that met the requirements was the largest at 1 m/s, followed by the 2 and 3 m/s conditions. The maximum Dent difference does not significantly vary for the three flight velocities, while the Cov varies considerably. For more samples, Cov was below 3% at 1 m/s, below 6% at 2 m/s, and mostly below 15% at 3 m/s. Additionally, although the Cov of some samples reached 30% at a velocity of 3 m/s, the Dent did not increase significantly, primarily due to the large size of the droplets at this velocity.

The effective coverage ratio (ECR) and effective density ratio (EDR) were calculated based on the aforementioned effective droplet deposition requirements (Table 2). The ECR at the flight velocities of 1 and 2 m/s were 73.3 and 71.1, respectively; however, it was significantly reduced at 3 m/s, which was 57.8 and 21.14% lower than that at 1 m/s. The EDR gradually decreased with an increase in the flight velocity, and the EDR was 48.90, 33.33, and 28.90% at the velocities of 1, 2, and 3 m/s, respectively. In terms of Cov within the canopy, the upper canopy coverage (Cov_Upp_), middle canopy coverage (Cov_Mid_), and lower canopy coverage (Cov_Low_) all exhibited an increasing trend with an increase in the flight velocity, such that the Cov_Mid_ at 1 m/s was 2.44%, and the Cov_Mid_ at 3 m/s increased to 6.49%. These results indicate that the ECR and EDR of the droplets decrease despite the increase in the flight velocity and the mean coverage (Cov_Mean_). Overall, an increase in flight velocity reduces the proportion of effective droplet coverage which meets pest control requirements.

The Cov at the three canopy heights did not significantly vary at different velocities. The interior zone coverage (Cov_Int_) and exterior zone coverage (Cov_Ext_) of the canopy are relatively similar, primarily due to the divergent canopy pattern of peach trees. Therefore, it is not as difficult to apply pesticide inside the canopy as that of dense fruit trees. Cov_Int_ reached 9.35 at a flight velocity of 3 m/s, which significantly exceeded the value of others. The individual samples were possibly contaminated during the application process. The Cov_Mean_ values at 1, 2, and 3 m/s were 2.13, 3.14, and 4.68%, respectively. The coverage distribution uniformity (CV) decreased with an increase in the velocity and the CV value increased from 74.7% at 1 m/s to 147.49% at 3 m/s. This is primarily attributed to the fact that the downward speed of the down-wash airflow is decomposed with the increase in flight velocity, and part of the airflow generates a wingtip vortex. Some droplets deviate from their initial motion direction under the action of a complex wind field, which can easily cause a sudden increase or decrease in the deposition in some canopy areas.

Dents exhibited opposite trends at different canopy heights (Table 2). The Dent value exhibited a gradual decreasing trend with the increase of the velocity. For example, the middle canopy droplet density (Den_Mid_) at 1 m/s decreased from 18.84 deposits/cm^2^ to 12.66 deposits/cm^2^ at 3 m/s, indicating a decrease of 32.80%. The Cov_Mean_ and mean droplet density (Den_Mean_) in the middle layer were higher than those in the upper and lower layers, which is related to the special downwash airflow auxiliary spray method of the UAV. The downwash airflow transports droplets to the inside of the canopy, and the upper canopy is most disturbed by the airflow due to the shaking effect of the branches and leaves. Some of the droplets cannot effectively attach themselves, while the lower part is more severely occluded by the middle and upper branches and leaves. The Den_Mean_ values at the three velocities did not exhibit a significant variation.

**FIGURE 7:**
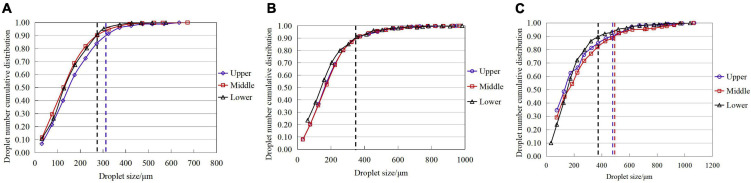
Cumulative ratio of droplet number and the corresponding droplet size at various canopy layers under different flight velocity. The vertical dotted lines represent the VMD when the cumulative proportion of droplet points reaches 0.9, where the blue, red, and black dotted lines represent the upper, middle, and lower layer of the canopy, respectively. **(A)** 1 m/s. **(B)** 2 m/s. **(C)** 3 m/s.

The cumulative ratio of the droplet number and the corresponding droplet size were calculated in 20 gradients (0–50 μm, 50–100 μm, 100–150 μm… 950–1000 μm), and the results are presented in Figure 7. The droplet number cumulative ratio curve at various canopy parts exhibits a high similarity at the same flight velocity. The droplet size varied at different flight velocities corresponding to a cumulative ratio equal to 0.9. At 1 m/s, the droplet size corresponding to the cumulative ratio equal to 0.9 in the middle and lower layers, was identical at 273 μm, while that in the upper layer was approximately 315 μm. At 2 m/s, the droplet size at the three canopy heights was consistent at 355 μm. For 3 m/s, the droplet sizes in the upper, middle, and lower layers when the cumulative ratio reached 0.9, were 475, 482, and 370 μm, respectively. In general, the droplet size at an accumulation ratio of 0.9 exhibited an increasing trend with the increase in velocity.

### Spray deposition characteristics of combined pesticides in the canopy

**FIGURE 8:**
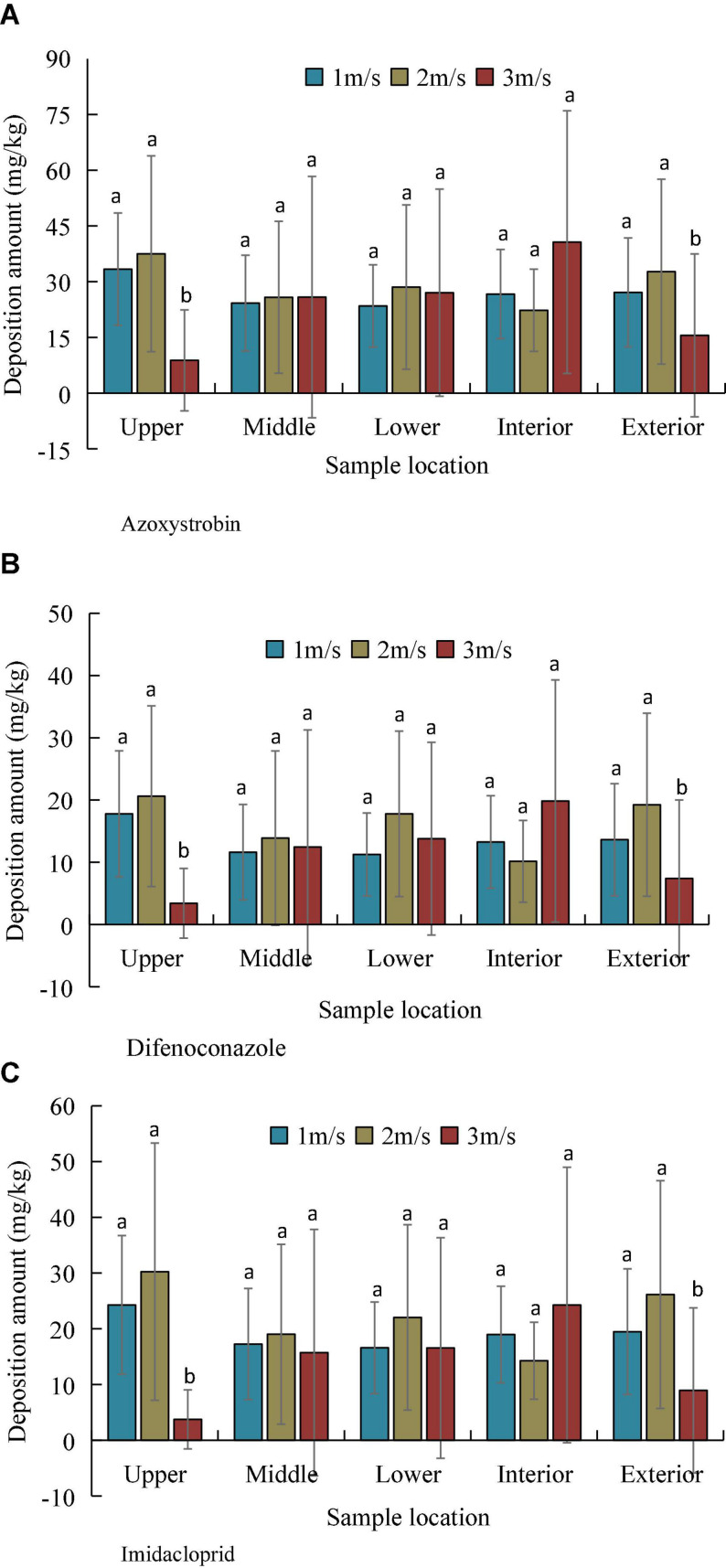
Deposition amount of pesticides at different velocities and canopy heights. **(A)** Azoxystrobin, **(B)** Difenoconazole, and **(C)** Imidacloprid.

The collected leaves were crushed, extracted, and filtered, and the difenoconazole, azoxystrobin, and imidacloprid contents were determined by using UPLC-MS/MS. The proposed method realizes trace detection of pesticide deposition and better reflects the pesticide attachment on the leaf surface when compared to the tracer method. We weighed the leaves at each sampling point due to the difference in the size of the collected leaves, to effectively characterize the amount of deposition on the unit leaves. Subsequently, we obtained the mass of all the leaves and obtained the deposition amount (mg/kg) of the unit mass. Figure 8 presents the experimental results, which demonstrate that the standard deviation of the amount of deposition is relatively large as a whole. This indicates that the deposition of the pesticide is very uneven, which corresponds to the trace detection method of the pesticide. The velocity significantly affected the deposition amount, and the average deposition was the lowest at a velocity of 3 m/s. Furthermore, we calculated the mean deposition of the three pesticides at different velocities (Table 3). The azoxystrobin mean deposition (DEP_Azo–Mean_) was the highest at 27.01, 30.61, and 20.56 mg/kg, followed by the Imidacloprid mean deposition (DEP_Imi–Mean_) and difenoconazole mean deposition (DEP_Dif–Mean_). The deposition amount was normalized owing to the differences in the dosages of the three pesticides during dispensation. The results demonstrated that the imidacloprid normalized deposition (DEP_Imi–nor_) was the highest, followed by azoxystrobin normalized deposition (DEP_Azo–nor_) and difenoconazole normalized deposition (DEP_Dif–nor_), which is mainly attributed to the precipitation of imidacloprid. In terms of dosage forms, azoxystrobin and difenoconazole are used as the suspension agents (SC) and imidacloprid is used as a wettable powder (WP). The pipe connected to the liquid pump is located at the bottom of the spray tank, and the content of the active ingredient of imidacloprid in the liquid can be increased at the bottom of the pipe or at the bottom of the tank even though the spray tank is shaken before the spray test to mix the liquid.

**TABLE 3:** Spray deposition distribution characteristics in canopy for the different flight velocities.

Parameters	Treatment			
		1 m/s	2 m/s	3 m/s
Difenoconazole vertical penetration efficiency	PE_VDif_ (%)	76.63	103.03	173.36
Azoxystrobin vertical penetration efficiency	PE_VAzo_ (%)	81.44	90.19	155.97
Imidacloprid vertical penetration efficiency	PE_VImi_ (%)	79.90	89.47	170.01
Difenoconazole horizontal diffusion efficiency	PE_HDif_ (%)	97.24	52.76	267.97
Azoxystrobin horizontal diffusion efficiency	PE_HAzo_ (%)	98.32	68.21	261.47
Imidacloprid horizontal diffusion efficiency	PE_HImi_ (%)	97.36	54.59	271.28
Difenoconazole deposition distribution uniformity	CV_Dif_ (%)	63.70	79.78	150.12
Azoxystrobin deposition distribution uniformity	CV_Azo_ (%)	51.95	75.57	130.00
Imidacloprid deposition distribution uniformity	CV_Imi_ (%)	55.28	80.20	149.91
Difenoconazole mean deposition	DEP_Dif–Mean_ (mg/kg)	13.55 ± 8.63ab	17.42 ± 13.90a	9.88 ± 14.84b
Azoxystrobin mean deposition	DEP_Azo–Mean_ (mg/kg)	27.01 ± 14.04ab	30.61 ± 13.13a	20.56 ± 26.69b
Imidacloprid mean deposition	DEP_Imi–Mean_ (mg/kg)	19.38 ± 10.71a	23.77 ± 19.06a	12.01 ± 18.00b
Difenoconazole normalized deposition	DEP_Dif–nor_ (mg⋅cm^2^/kg⋅μg)	10.84 ± 6.91ab	13.94 ± 11.12a	7.91 ± 11.88b
Azoxystrobin normalized deposition	DEP_Azo–nor_ (mg⋅cm^2^/kg⋅μg)	13.52 ± 7.02ab	15.30± 11.57a	10.28± 13.34b
Imidacloprid normalized deposition	DEP_Imi– nor_ (mg⋅cm^2^/kg⋅μg)	25.83 ± 14.28a	31.69 ± 25.41a	16.01 ± 24.00b

The deposition distribution characteristics of the pesticide solutions were analyzed at different locations in the canopy. The results demonstrated that an increase in the flight velocity improved the penetration efficiency of the pesticide solution. For example, the azoxystrobin vertical penetration efficiencies (PEV_Azo_) at 1, 2, and 3 m/s were 81.44, 90.19, and 155.97%, respectively, and that at 3 m/s was 91.51% higher than that at 1 m/s. Similarly, the lateral horizontal diffusion efficiency of the canopy also improved. The horizontal diffusion efficiency of azoxystrobin (PEH_Azo_) increased from 98.32% at 1 m/s to 261.47% at 3 m/s. However, a higher flight velocity increases the coefficient of variation of the droplet deposition distribution, due to which the deposition distribution becomes uneven and the azoxystrobin deposition distribution uniformity (CV_Azo_) reaches 130% at 3 m/s.

### The effect of flight velocity on ground loss

**FIGURE 9:**
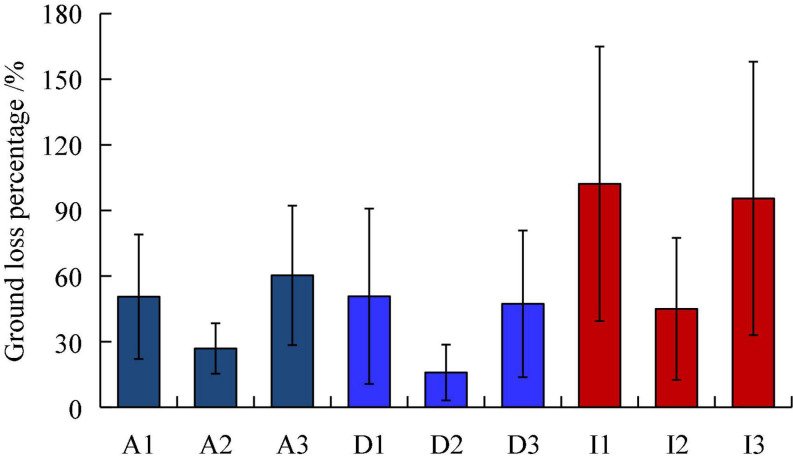
Histogram of ground loss percentage of three pesticides at three flight velocities. On the abscissa, the letters A, D, and I denote Azoxystrobin, Difenoconazole, and Imidacloprid, respectively. The numbers 1, 2, and 3 denote the flight velocity of 1, 2, and 3 m/s, respectively.

The effects of different flight velocities on the ground loss of pesticides under tree canopies were analyzed. The ground loss value of the three pesticides was minimum when the flight velocity was 2 m/s, and the deposition loss on the ground was similar at velocities of 1 and 3 m/s, as shown in Figure 9. This is because the droplets are driven to move toward the canopy by the downwash flow when the wind velocity is 1 m/s, and some droplets drop from the leaf surface or are directly deposited on the ground through the canopy gap. The downwash airflow velocity decomposed at a flight velocity of 3 m/s, and the fine droplets moved along a zig-zag direction to the ground under the action of the wing tip vortex, resulting in a large pesticide loss.

### The effect of flight velocity on spray drift

**FIGURE 10:**
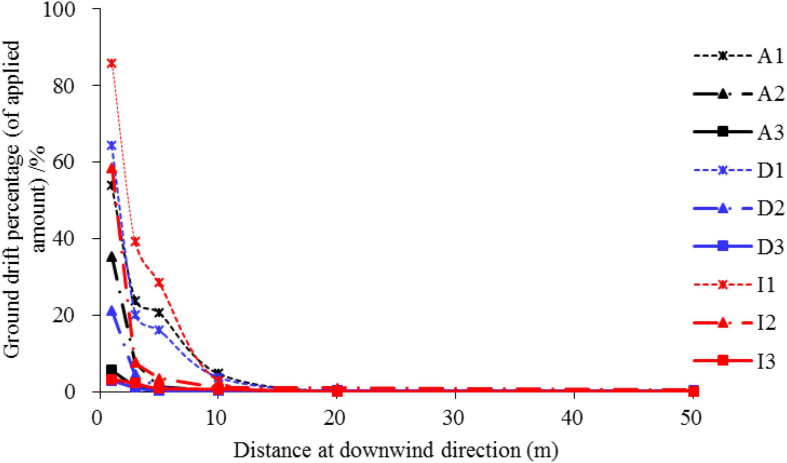
The curve of downwind ground drift at the setting distance from the spray treated area. The letters A, D, and I represent Azoxystrobin, Difenoconazole, and Imidacloprid, respectively. The numbers 1, 2, and 3 represent the flight velocity at 1, 2, and 3 m/s, respectively.

Figure 10 depicts the pesticide drift curve within 50 m of the drift treatment area. The drift volume exhibits a gradual decreasing trend with an increase in the flight velocity. This concurs well with the existing reports (Chen et al., 2020). The drift percentage can reach 85% at 1 m, and the drift percentage is less than 5% when the distance is 10 m. The drift percentage was less than 0.2% 50 m downwind (Table 4).

**TABLE 4:** Spray drift percentage (%) for the three pesticides used in the experiments under the setting flight velocity.

Drift percentage (%)		Azoxystrobin			Difenoconazole			Imidacloprid		
		1 m/s	2 m/s	3 m/s	1 m/s	2 m/s	3 m/s	1 m/s	2 m/s	3 m/s
Distance from the spray treated area (in m)	1	53.7930	35.2925	5.5225	64.3002	21.0757	2.9056	85.8106	58.3701	3.2498
	3	23.8631	7.6444	1.5557	20.0874	4.4341	1.0670	39.2380	7.5472	2.1489
	5	20.6228	1.3623	0.3638	16.0613	1.0287	0.2478	28.5091	3.5206	0.8438
	10	4.7109	0.2953	0.2952	3.6673	0.1931	0.2364	2.8036	0.9102	0.6067
	20	0.0094	0.1613	0.2142	0.0384	0.1366	0.1820	0.0236	0.8959	0.0692
	50	0.0081	0.1422	0.1313	0.0145	0.1189	0.1729	0.0024	0.4365	0.0510
	Average	17.1679	7.4830	1.3471	17.3615	4.4978	0.8019	26.0645	11.9467	1.1615
Sampling height at 100 m distance from the spray treated area (in m)	2	0.0019	0.0708	0.4194	0.0072	0.0392	0.1423	0.0073	0.2016	0.9993
	3	0.0022	0.0809	0.5098	0.0186	0.0167	0.2735	0.0112	0.1240	1.0283
	4	0.0019	0.1005	0.4406	0.0068	0.0471	0.1873	0.0012	0.1428	0.7536
	5	0.0000	0.0602	0.2631	0.0076	0.0523	0.0623	0.0099	0.0699	0.3878
	6	0.0043	0.1436	0.3329	0.0262	0.0540	0.0832	0.0006	0.2900	0.6540
	7	0.0011	0.0958	0.3590	0.0043	0.0361	0.1426	0.0015	0.1716	0.5961
	8	0.0011	0.0715	0.6266	0.0051	0.0592	0.2888	0.0015	0.1581	0.9262
	9	0.0048	0.0943	0.4399	0.0071	0.0240	0.1541	0.0519	0.1431	0.6550
	10	0.0023	0.0484	0.0626	0.0146	0.0264	0.0253	0.0016	0.1348	0.1223
	Average	0.0021	0.0851	0.3837	0.1083	0.0394	0.1510	0.0096	0.1595	0.6803

**FIGURE 11:**
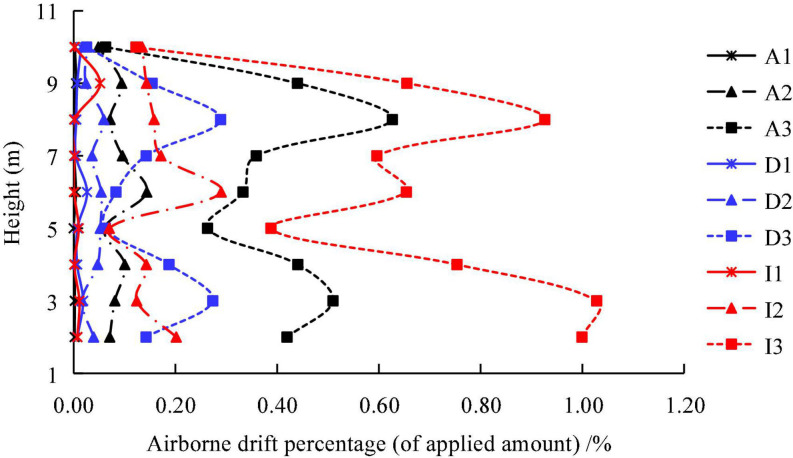
Airborne drift profile at 100 m distance from treated area. The letters A, D, and I represent Azoxystrobin, Difenoconazole, and Imidacloprid, respectively. The numbers 1, 2, and 3 represent the flight velocity at 1, 2, and 3 m/s, respectively.

The pesticides can still be detected 100 m downwind, and the drift percentage goes up to 1%, indicating that the application drift of the plant-protection UAV remains relatively significant. Figure 11 presents the drift percentages at different heights. Overall, the pesticide drift in the vertical direction was not closely related to the height. However, the vertical distribution of the three pesticides was saddle-shaped at 3 m/s, and the drift volume was highest at 3 and 8 m. The airborne drift was the highest at 3 m/s when compared to the three flight velocities, which was the opposite of the close ground drift (the drift volume was the highest at 1 m/s at the three flight velocities). This is primarily attributed to the increase in the flight velocity, which forms a characteristic airflow structure such as a wingtip vortex. These vortex structures typically exhibit higher energy, and the fine droplets can be transported in the air over a long distance under the joint action of the ambient crosswind. Among the three pesticides, imidacloprid presented the highest drift percentage, followed by azoxystrobin, while difenoconazole presented the lowest drift percentage, which was consistent with canopy normalization deposition.

## Discussion

In recent years, the use of plant protection UAVs for pesticide spraying operations has been increasing rapidly in China. The application field has gradually extended from grain crops to commercial crops such as fruit trees, tea trees, and vegetables. This operation method does not consider topographic factors, and thus presents broad development and application potential in hilly areas where it is difficult to operate ground machinery. The plant protection UAV presents the characteristics of low water consumption and high liquid concentration when compared to the traditional spraying method. Multiple pesticides can be simultaneously included in a single spraying process, which has been widely implemented to avoid various pests and diseases. For example, the application strategy of “one spraying and three defenses” is implemented for wheat when using the plant-protection UAVs in China. A similar strategy was also used for orchards. Therefore, we conducted spray tests of a plant-protection UAV using a combination of three pesticides in a peach orchard. The effect of flight speed on the droplet size, deposition in the canopy, ground loss, ground drift, and airborne drift was analyzed.

Firstly, the effect of flight velocity on the volume median diameter was analyzed (Figure 4). The results demonstrate that the flight velocity significantly affects the droplet size. A higher flight velocity increases the droplet size on the target, which is attributed to the down-wash airflow generated by the UAV rotor wing. An increase in the flight velocity contributes to the formation of wingtip vortices and other airflow structures. These vortex structures cause the fine droplets to spread with the airflow, resulting in the deposition of larger droplets on the canopy. It was also observed that the droplet size in the lower canopy was slightly smaller than those in the middle and upper parts.

We also analyzed the diffusion ratio (RD) of the dyed droplets on a water-sensitive paper surface (Figure 5), which is typically used to characterize the uniformity of the droplet size distribution. The droplet size distribution was not ideal, and failed to meet the spray requirement of RD > 0.67. Xu et al. (2017) conducted a spray test using a plant-protection UAV in rice, and the obtained RD value did not exceed 0.67. This is mainly attributed to the combined effect of the plant canopy and UAV downwash airflow. The canopy foliage blocks larger droplets from deposition, and the downwash airflow drives smaller droplets away from the initial trajectory. Computer simulation modeling must be employed to further analyze the diffusion law of droplets with different sizes inside the canopy to optimize the nozzle and flight parameters and achieve a uniform distribution of droplet sizes.

The droplet coverage characteristics of the water-sensitive paper were measured. A good linear correlation was observed between the droplet coverage and droplet density since the droplet coverage is lower than 20% (Figure 6), which is consistent with the findings of Grella et al. (2022). Wang et al. (2022) selected a droplet density higher than 15 deposits/cm^2^ and a coverage rate higher than 1% as the lowest threshold indicators and analyzed the spray coverage parameters. For the three flight velocities considered in the experiment, the number of samples that met the requirements was largest at a speed of 1 m/s, followed by 2 and 3 m/s. This implies that increasing the flight velocity can reduce the control effect when the application rate is fixed. Consequently, the effective coverage ratio (ECR) and effective density ratio (EDR) were calculated at different speeds (Table 2); they tended to decrease with an increase in the flight velocity. Furthermore, we observed that the average droplet coverage rate gradually increased with the increase in the flight velocity, and the average coverage rates at 1, 2, and 3 m/s were 2.13, 3.14, and 4.68%, respectively, which was not consistent with the expected values. This is mainly attributed to the increase in the droplet size as explained earlier. Blue streaks were observed on the surface when processing the water-sensitive paper, which indicate the “hard landing” of a droplet. That is, the droplets from UAV applications may impact the target surface with significant high-speed horizontal motion (Li et al., 2021a). This was mainly attributed to the combined action of forward flight inertia and downwash airflow, which extended the dyed area.

The droplet density gradually decreased with the increase in the flight velocity, contrary to the droplet coverage. This is because the droplet density measurement does not consider the droplet point area in the statistics as long as the dyed droplet point exists individually. Therefore, the number of droplets did not increase at a flight velocity of 3 m/s despite the increase of the droplet coverage rate. In general, the droplet coverage rate and density in the middle canopy were higher than those in the upper and lower canopies, which was primarily attributed to the disturbance of the upper canopy due to airflow and the occlusion of the lower canopy due to branches and leaves.

The droplets on all the water-sensitive paper samples were statistically analyzed. The droplet size was divided into 20 gradients with an interval of 50 μm in the range of 0–1000 μm. The cumulative ratio of the droplet numbers under different operating conditions was calculated (Figure 7). The droplet size corresponding to the droplet number cumulative ratio value of 0.9 increases with the increase in the flight velocity. At 2 m/s, the corresponding droplet sizes of the upper, middle, and lower canopies were observed to be identical when the cumulative ratio was 0.9. The droplet sizes for the three canopy heights varied at 3 m/s.

For the pesticide deposition in the canopy, we used UPLC-MS/MS to determine the active ingredient amounts of the three pesticides on the leaves in different parts of the canopy. The flight velocity significantly affects the pesticide deposition. The mean deposition amount was highest at 2 m/s and lowest at 3 m/s. Zhang et al. (2012) reported that the deposition amount negatively correlated with the flight velocity of rice sprayed with an unmanned helicopter. In this study, the flight velocity significantly affects the uniformity of deposition. Therefore, an increase in the flight velocity is detrimental to uniform deposition. The CV reduced from 60% at 1 m/s to 140% at 3 m/s, which is consistent with the findings of Qiu et al. (2013). However, Chen et al. (2016) reported that the flight velocity significantly affects the deposition amount, but does not significantly affect the deposition uniformity. This deviation in the experimental conclusions is primarily attributed to the differences in the target plant and the types of plant protection UAVs used. Among the three pesticides used in this study, the normalized deposition amount of imidacloprid was the highest, indicating that it had the best deposition efficiency, followed by azoxystrobin and difenconazole. This phenomenon may be attributed to the fact that the dosage form of imidacloprid is a wettable powder (WP), which tends to accumulate at the bottom of the tank during operation. It was observed that an increase in the flight velocity improved the vertical penetration efficiency (PEV) and horizontal diffusion efficiency (DEV) while causing an uneven deposition distribution.

The ground loss percentage of the applied amount under the canopy was relatively large in terms of the pesticide loss and spray drift (Figure 9 and Table 4), particularly for the imidacloprid component, which reached a maximum of nearly 100%. For the three flight velocities, the ground loss percentage was the lowest at 2 m/s, and the deposition amount and normalized deposition amount were the highest at this speed, indicating that a better deposition effect was achieved at a 2 m/s flight velocity. The ground loss in vineyards with UAV spraying were studied by Biglia et al. (2022), and found that the ground losses decrease with the increase of the UAV cruise speed. This difference is mainly caused by the canopy morphology, planting pattern of fruit trees and UAV operation mode. The ground drift percentage gradually decreased with the increase in the distance from the spraying-treated area, and the drift percentage was lower than 5% when the distance was 10 m, this is consistent with the results obtained by Wang et al. (2021). The sprayed pesticide could still be detected in the air at 100 m downwind, and the airborne drift percentage of imidacloprid reached 1% at a height of 2 m under 3 m/s. In general, the airborne drift is larger at the heights of 3 and 8 m, which makes the distribution curve appear saddle shaped. At present, the aerial drift of plant protection UAV sprays is mainly measured in the near field within 20 far away from the spray area (Wang Z. C. et al., 2020; Wang et al., 2021). Wang G. B. et al. (2020) tested the airborne drift at 12 m from the sprayed erea, and found that at a height of 1–5 m from the ground, spray drift gradually decreased with the increase of height.

## Conclusion

In this study, the effects of flight velocities on the droplet size, deposition distribution in the canopy, ground loss, and spray drift of peach orchards were systematically analyzed to improve the spray effect. The flight velocity significantly affects the droplet size, and an increase in the flight velocity increases the droplet size on the target. The droplet size in the lower canopy was slightly smaller than those in the middle and upper parts. Unfortunately, the diffusion ratio of the droplets is not ideal and is not greater than 0.67. A higher flight velocity presented a larger droplet coverage rate and a smaller density on the target, which was mainly attributed to the expansion of the dyed area formed by the hard landing of the droplet. The increase in the flight velocity reduced the effective coverage ratio and effective density ratio, while increasing the vertical penetration efficiency and horizontal diffusion efficiency; however, it also reduced the uniformity of the droplet deposition distribution. The ground loss and spray drift were significantly high during the operation of the plant protection UAV, and the maximum airborne drift percentage reached 1% at a distance 100 m away from the spraying treated area.

This study quantified the deposition and drift of pesticides from the plant protection UAVs sprayed at different flight velocities based on the analysis of ultra-high-performance liquid chromatography-tandem triple quadrupole mass spectrometry, which provides a reference for the commercial application of plant-protection UAVs. However, several aspects must be determined to determine the effect of other parameters such as flying height and spraying dosage on the spray effect in order to improve the spray performance of the plant-protection UAVs. The actual performance of peach trees must be evaluated for pest control before the commercialization of the optimized operation strategy when compared to the traditional manual knapsack method. Additionally, the residue and digestion dynamics of pesticides in fruits must be further analyzed after the plant-protection UAV spraying.

## Data availability statement

The original contributions presented in this study are included in the article/supplementary material, further inquiries can be directed to the corresponding authors.

## Author contributions

LL: conceptualization, methodology, and writing – original draft. ZH: data curation, formal analysis, writing – review and editing, and funding acquisition. QL: software, data curation, and investigation. TY: resources, validation, and investigation. PH: resources, writing – review and editing, and funding acquisition. RZ: methodology, supervision, writing – review and editing, and funding acquisition. LP: methodology. All authors contributed to the article and approved the submitted version.
